# A Mixed Methods Exploration of Social Media Use for Health Information in Under-Resourced Communities

**DOI:** 10.3390/ijerph22071081

**Published:** 2025-07-06

**Authors:** Nishita Matangi, Maud Joachim-Célestin, Cristie Granillo, Valeria Rodarte, Beverly Buckles, Theresa Ashby, Nikhil Thiruvengadam, Susanne Montgomery

**Affiliations:** 1 School of Behavioral Health, Loma Linda University, 11065 Campus St, Loma Linda, CA 92350, USA; cgranillo@llu.edu (C.G.); bbuckles@llu.edu (B.B.); 2 School of Behavioral Health Interdisciplinary Studies, Loma Linda University, 1686 Barton Rd., Redlands, CA 92373, USA; mcelestin@llu.edu (M.J.-C.); nthiruvengadam@llu.edu (N.T.); 3 Department of Preventive Medicine, Loma Linda University School of Medicine, 11175 Campus Street, Loma Linda, CA 92350, USA; 4 Institute for Community Partnerships, Loma Linda University Health, 11188 Anderson St, Loma Linda, CA 92354, USA; vrodarte@llu.edu; 5 School of Public Health, Loma Linda University, 24951 Circle Dr, Loma Linda, CA 92354, USA; tashby@llu.edu; 6 Division of Gastroenterology and Hepatology, Loma Linda University, 11234 Anderson St, Loma Linda, CA 92354, USA

**Keywords:** health communication, health literacy, social media, digital literacy, health inequities, organizational health literacy, under-resourced communities, mixed methods

## Abstract

Social media (SM) use and the burden on healthcare systems have concurrently increased, with the latter resulting in longer wait times and higher costs. As a result, more people seem to use social media to access health information (HI). This study explores how SM is used for accessing HI within an under-resourced community. In this mixed methods study, respondents (N = 256) completed online English and Spanish Qualtrics surveys assessing their use of healthcare services and social media, and its use for HI. We also explored respondents’ experience in understanding and using the HI they found on SM. Qualitative inquiries (N = 7) included focus groups and key informant interviews and expanded on the survey results. Results indicated that most participants used SM for HI. Instagram, Snapchat and TikTok were associated with looking up HI before and after receiving care and for health decision-making and for considering treatments or medication after seeing information about these on social media. To create effective messaging that is accepted, relatable and easy to access for the audiences they seek to reach, healthcare organizations must understand how SM is used as a source of HI. Exploring the associations between SM algorithms, health literacy, access to healthcare and SM use can help improve health communication strategies to be used on SM platforms.

## 1. Introduction

Raising awareness and providing critical and timely information about relevant health issues is an important factor in promoting health behavior change and consequently leads to health improvements [1]. Thus, health information and the public’s ability to understand it (health literacy), is increasingly considered to be one of the determinants of health [2,3,4]. Although interest in understanding how to effectively disseminate clear and accurate health information peaked during the COVID-19 pandemic crisis, in the professional community and in society at large, this interest is not new.

With the advent of the internet and social media, communication strategies and media have dramatically changed, requiring major adaptations and updates in dissemination approaches [5,6]. As a result, healthcare institutions have attempted to broadcast health information to the public by using different media, including social media platforms. Questions remain, however, as to the effectiveness of such media use, especially among those most affected by health disparities.

Health disparities or inequities have always existed and are impacted by factors such as race, ethnicity, location, socioeconomic status and other demographic variables that affect specific populations [7]. While health status in the United States (US) has improved for most, preexisting gaps in health outcomes between various groups have mostly widened [8].

San Bernardino County, California (SBC) is an example of a community that experiences extensive health and social disparities. According to data from the California Health Interview Survey, 13% of SBC young adults are uninsured and 15.8% of SBC residents over the age of 65 delayed obtaining medical care or did not receive the care they needed. SBC has a less than favorable ratio of 1710 residents to each primary care physician, has 49 healthcare provider shortage areas and ranks 56 of 58 California counties in quality of clinical care. Together these factors contribute to disproportionate rates of delayed access to care, leading to poor health outcomes for residents of this county [9].

Populations affected by social and health disparities often have a lower education level, which results in lower health literacy contributing to health disparities. Lower health literacy is a barrier to understanding the complex issues around health and health decision-making. Thus, it is associated with lack of involvement with health decision-making, poorer patient-provider communication, less adherence to treatment and medication, difficulties with discharge instructions and higher rates of hospitalization, readmissions and emergency care. This not only leads to an even higher burden for individuals but is increasingly costly to healthcare systems [10].

Indeed, information that is difficult to access, navigate and understand can result in costly consequences not only to the individuals but to the organizations tasked with serving them [11]. This is especially true in an increasingly burdened healthcare system: a survey conducted in 2022 found that the average wait time for a physician appointment was 26 days, which is an 8% increase from 2017 and a 24% increase from 2004 [12]. The U.S. Healthy People 2030 distinguished organizational health literacy from personal health literacy in which organizations are held responsible for providing health information that is easy for their respective communities to find, understand and use [13]. This conceptual framework—Organizational Health Literacy (OHL) was a key guide in the approach and methodology of this study.

Previous research has shown that healthcare organizations are not as responsive to low health literacy populations as they could be [11,14,15]. However, with the increasing burden of disease, the need to create content that is culturally sensitive and accessible [3,13,16] has become more urgent. Since their inception, public institutions considered to be credible or trusted sources (TS) such as the World Health Organization (WHO), the Center for Disease Control and Prevention (CDC) and the National Institutes of Health (NIH) [2,17,18] have played an important role in determining health outcomes by improving access to accurate and evidence-based information with the public. To address health disparities, these and other health organizations are expected to create both accurate health information content and content that is accessible and understandable to everyone, including those in disadvantaged communities [17].

Partially shifting the burden of being a ‘good’ consumer of health information from the patients to the organizations that serve them [13] represents a shift in the responsibility to fill the informational gaps. This approach is in keeping with effective communication strategies, which increasingly place the receiver at the center of the message and have been successfully applied in the distribution of health information [3,11].

The number of people using social media (SM) as a source of information is increasing. In 2023, an estimated 4.9 billion people used SM globally, which is projected to increase to 5.85 billion by 2027 [19]. It is also well established that SM is increasingly becoming the primary source of news and information, including health information for most Americans [20]. The average user spends about two and a half hours across multiple SM platforms [21].

Because SM has impacted society as a whole and changed the way we interact, obtain, share and process information, more health information is now available on SM. This became especially evident during the COVID-19 pandemic. SM is increasingly used for health communication specifically among adults living in the USA [22]. An estimated one-third of adults in the USA use the internet to either learn about a health concern or even to self-diagnose or co-diagnose on an issue [23].

According to principles of effective communication, the more relatable the content is to an individual, the more likely that person is to interact with it and/or to act upon the information provided. This is particularly relevant and consequential in the case of social media (SM) because dissemination of content in this setting is often subject to factors beyond the control of the producer such as the platform’s algorithms, which vary based on the level of engagement. Even if health organizations practice good OHL in the content they create for their audiences, if they fail to take into account the current algorithm of the SM platform they are using, their reach is negatively affected. A lack of understanding of trends, post formats, timing and other factors can impact if and how the health information they share reaches their audiences.

Indeed, although increasing use of SM by the public for health information has made it easier to share information on a large scale, effective health communication has become more complex and difficult due to the moderating factors of algorithms, politicization and peer interaction which can create echo chambers [24] and reduce access to potential viewers. The Social Exchange Theory suggests that individuals maximize the benefits over the costs of social exchanges [24,25,26]. In the world of social media, the concept of cost can be translated into an individual’s time spent stopping on a post when scrolling through their feed against the information that they gain from a post. Moreover, the way algorithms influence what one is exposed to, is dependent on a user’s time spent on a post (e.g., an organization’s post); thus, the more time users spend on a post, the greater the reach that post will have. This in turn will also increase the probability of the user receiving posts that are similar to the ones they have spent time on.

As a result of this engagement-driven nature of SM, health professionals have been hesitant to use SM as a form of health communication, especially due to the potential lack of accountability and academic rigor inherent in its use. Yet, given the reality of the public’s increased use of SM to acquire health information, and in accordance with best communication strategies, it is incumbent upon trusted sources to engage effectively with SM to reach their audiences. It is also important for health organizations to be proactive rather than reactive with the content they produce, especially in times of crises [25].

In this context, there has been an increase in the presence of hospitals, disease centers, clinics and other health organizations, like the WHO and CDC on various SM platforms, especially during and after epi- and pandemic times like COVID-19, Ebola and H1N1 [22]. Some have even developed personalities within the social media space with an individual or mascot often being the face of the account [26].

It is not clear, however, if health information is distributed and accessible to the intended audiences, including those in under-resourced communities. Indeed, some individuals, such as uninsured, low-income, lower-educated population may have a greater need for accessible health information on SM. Rawls’ Distributive Justice Theory encourages fair distribution of goods and resources which in this case can include health information [27]. One of the aims of aim of OHL, is to determine if content is being produced in a way that encourages those consumers to interact with the messages intended for them [11]. For that to happen, we need to know which virtual platforms people from under-resourced communities use and understand their patterns of use. To explore these issues in the context of one US County, San Bernardino County, the current study assessed the use of SM in a representative section of SBC. We hypothesized that SBC residents actively use SM in some capacity to access health information. In addition, when evaluating the OHL of credible sources on social media, we predicted there would be some disconnect between the available organizational health communication and what the community seeks and considers accessible.

## 2. Materials and Methods

**Figure 1:**
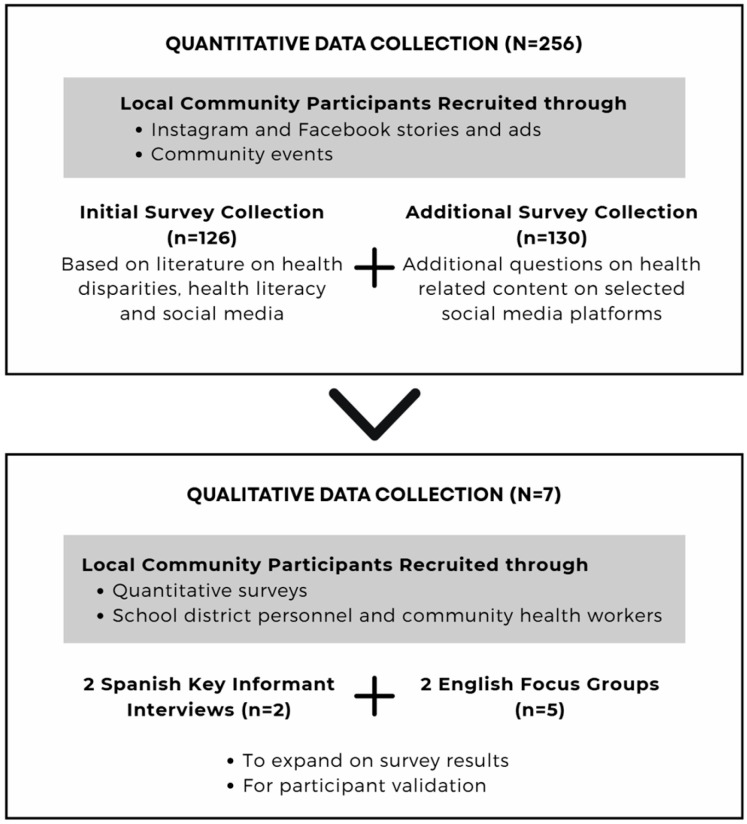
Flow chart depicting participant recruitment and data collection.

This mixed methods study included a quantitative assessment and data collection (N = 256) followed by qualitative input (N = 7) for a better understanding of quantitative results as well as participant validation (member feedback) with members from the community of interest (see Figure 1). Procedures were approved by the Loma Linda University Institutional Review Board (#5240309).

### 2.1. Participants and Procedures

Inclusion criteria for both study portions were that participant be (1) 18 years or older and (2) a current resident of SBC. For the quantitative portion of the study, participants were recruited through social media stories, social media post advertisements and links shared by local organizations as well as in-person events (community health and resource distribution). After signing a virtual embedded informed consent, participants completed surveys over a 5-month period. Surveys were available in the participant’s preferred language (English or Spanish).

Because this survey was intended for individuals who already regularly use social media, it was digitally administered through Qualtrics and was designed to be responsive and mobile-friendly. Because of the novelty of this study, to our best knowledge, there were no known standardized instruments for the survey. However, the survey was designed based on an extensive literature review from different disciplines related to health disparities, health literacy and social media use in general. Besides demographic information, the survey explored perceptions, attitudes and practices of respondents’ general use of social media, as well as their use of social media for health information [28], and delays/barriers in accessing healthcare. For a subset of participants, and to have a more in-depth understanding of the patterns, we also obtained additional information about their social media use (social media platforms/accounts and types of use) and health information gathering/decisions in relation to the accounts they used. As compensation for their time and effort, those who completed the survey were given the option of entering a $25 gift card drawing.

The qualitative portion of our study was conducted to allow us to further explore and discuss issues identified in the survey and for participant validation (check-in). Participants were therefore recruited among those who completed the survey and were given the choice to join either a Spanish or English focus group (FG) discussion or be interviewed via Zoom, one month after the survey collection period. Although there were over 25 participants interested in participating in a focus group one English focus group had 2 participants and the other had 3. There were 2 Spanish-speaking participants, who requested one-on-one interviews for scheduling convenience and comfort instead of participating in a group setting (focus group discussion) as initially planned. It is important to note that qualitative data collection took place in early 2025, when there was much fear in the Latino communities due to political uncertainty related to immigration, especially in SBC where 21.4% identify as foreign-born [29].

After being briefed about the purpose of the discussion and guided through the consent process, participants confirmed their consent to participate and be recorded via Zoom. They also completed a short demographic form. Each discussion lasted approximately one hour. All discussions were facilitated by a trained and IRB-certified researcher—a native Spanish speaker in the case of the Spanish key informant interview (KII). Interviews were conducted using a semi-structured guide, and were audio and video recorded. Questions explored social media use in general, social media use for health information and feedback about two influenza (flu)-related social media (Instagram) posts.

### 2.2. Measures, Data Management and Analysis

G*Power 3.1 software [30] was used to determine the necessary sample size for statistical power. The alpha level was set to 0.05 and desired power was set to 0.9. For a two-tailed, bivariate normal model correlation of 0.3 and a null hypothesis correlation of 0, the final sample size was sufficient for statistical power for all analyses performed, allowing for a deeper inquiry into different types of social media usage by participant group.

Data from the Spanish and English surveys were combined into one master data set, which was managed and cleaned in Google Spreadsheets and split into two data sets. The overall data set included all participants and was used for descriptive statistics. However, after initial analysis it was evident that, to have a comprehensive understanding of the issue, knowing which platforms were used needed to be included. We therefore expanded data collection asking participants additional questions about what social media platforms they used. This subset (N = 130) was then used to further analyze the relationship between social media use and health information and met the criteria for based on our correlational power analyses. Bivariate correlation analyses were conducted in R using the Julius AI platform. Variables were organized into several categories: demographic, personal healthcare, social media use, health information seeking behavior and feedback on a specific social media message. Because this is a novel, emerging field and our study was designed to be an exploratory, we sought to keep the survey brief and mainly correlational, which affected our ability to explore more complex models.

Demographic variables included age, gender, education (less than high school, completed high school or equivalent such as General Equivalency Diploma/GED, certificate or trade school, bachelor’s degree or graduate degree), employment (unemployed, employed part or full time, working 2 or more jobs), preferred language (Spanish or English) and whether they were a parent/caretaker (Par/Car) or not. Personal healthcare variables included healthcare insurance type—no insurance, public/government or private—and whether participants attended a doctor’s visit in the past year (Health Care), had a primary care physician (PCP), waited 2 or more weeks for a healthcare appointment (Appt wait), had a delay in seeing a healthcare provider due to financial reasons ($ Delay) and whether or not the participant had been diagnosed with a chronic condition (Chronic Disease). Response options for the last four variables were “yes”, “no” and “maybe”.

Social media use variables inquired about number of social media platforms used, frequency of use, types of social media platforms used (X, Snapchat, WhatsApp, TikTok, Instagram or Facebook) and purpose of social media use—whether to connect with family and friends, to view the news, for entertainment or for pop culture (trending fashion, music, movies, recipes, etc.). Participants were also asked if they engaged most with or “followed” a person they considered a respected and knowledgeable health figure (“influencer”), a health organization, a health community or another type of health account.

For the qualitative data collection, health information seeking behaviors were assessed by asking participants if they used social media to search for health information, how easy it was to find health-related information, relatability, cultural relevance and perceived ease of understanding of the content. Participants were also asked if they had made a health decision based on the information they saw on social media, and if they felt that it had helped them make health decisions, such as considering the use of a specific treatment or medication. Lastly, participants were asked if they had spoken to their healthcare provider about the health information they had seen on social media, and whether health information searches had occurred after a medical appointment.

Qualitative discussions were transcribed with participant consent—and translated into English if recorded in Spanish—and then verified against the audio-recordings to check for accuracy. High-level thematic analyses were performed in MaxQDA [31]. Participants also completed a brief demographic form assessing their education level (no high school, high school graduation or GED, bachelor’s or graduate degree), their racial and ethnic identity (White, Black or African American, American-Indian or Alaska Native, Asian, Hispanic/Latino) and what social media platforms they used (Facebook, Instagram, TikTok, X, WhatsApp, Snapchat, YouTube, Reddit, Pinterest, LinkedIn or Threads). Each transcript was coded inductively, and codes were then categorized into themes. All codes and outputs were verified by the researcher to check for any errors or misinterpretation.

## 3. Results

### 3.1. Quantitative Results

A total of 256 participants completed the entire online survey. Baseline demographics and healthcare-related information can be found in Table 1. Demographic results were based on analyses from all 256 participants. SM-related descriptive results and bivariate correlation results were from the subset of 130 participants.

### 3.2. Descriptive Results

While the participants’ age ranged between 18 and 78, the average age was 34 years old (standard deviation = 20.97). A majority (80.2%) chose English, more than half (61.9%) reported being a parent or caretaker, 39.7% had no more than a high school degree or equivalent and 31.91% were unemployed. Most (87.6%) had some form of health insurance coverage, and 37.7% reported having public health insurance. When asked about their healthcare, 82.1% reported visiting a doctor in the past year, 68.9% had a primary care physician and only 28% reported having a diagnosed chronic health condition. Regarding healthcare appointments, 74% of participants had to wait at least 2 weeks for an appointment and 43.2% attributed the delay to financial reasons (see Table 1).

**Figure 2:**
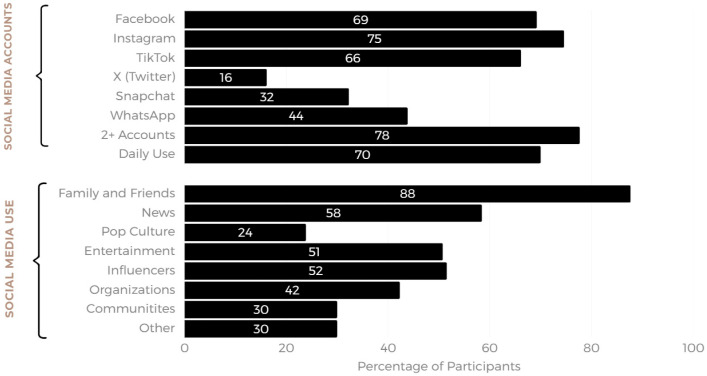
Overview of preferred social media platforms and types of use.

The SM platforms used the most were Instagram and Facebook (74.6% and 69.2% of participants, respectively), followed by TikTok (66.2% of participants). Regarding frequency and purpose of use, 70% reported daily use and most (87.7%) reported using the platform to follow friends and family, while 58.5% used it to access the news. When asked about the types of accounts they used, most followed “influencer” accounts (51.5%), followed by 42.3%, who followed posts by organizations (see Figure 2).

**Figure 3:**
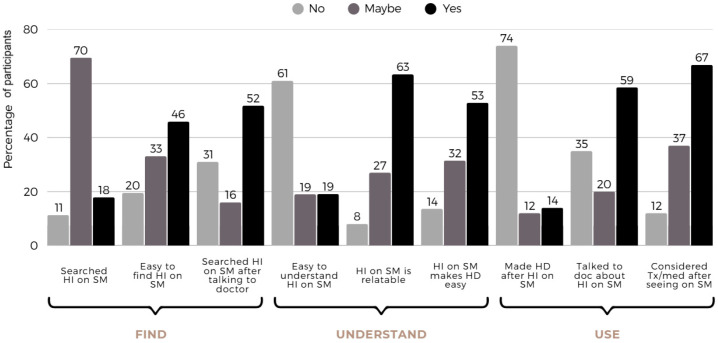
Social media use for health information. (HI—Health information; SM—social media; HD—health decision).

Participants were also asked about their experiences and behaviors around finding health information on social media (see Figure 3). While only 17.9% reported using SM to search for health information (HI), 69.6% were not sure whether or not they had done so, while only 11.3% did so intentionally. Nearly half (45.9%) said they thought it was easy to find HI on SM in contrast to 19.5% who did not. Although 64.4% stated that the health information they saw was relatable and culturally relevant, the majority (61.1%) did not find it easy to understand. When asked about the timing of social media use in relation to healthcare visits, 51.8% reported looking up health information on social media after talking to their doctor and more than half (58.6%) reported having talked to their doctor about health information they had seen on social media. While only 14% admitted to having made a healthcare decision after watching health information on social media, as many as 52.9% said the information they saw helped them make a health decision. Even more (66.9%) said they had considered using a treatment or medication after viewing something on social media.

### 3.3. Bivariate Correlation Results

Table 2 describes the associations between social media use by platform, reason for use, types of accounts followed and demographic variables. Language was statistically significantly associated with choice of platform: those who spoke Spanish were less likely to use X (Twitter), r (130) = −0.020, *p* = 0.021, but more likely to use WhatsApp, r (130) = 0.287, *p* = 0.001. Females were statistically significantly less likely to report the use of Instagram, r (130) = −0.207, *p* = 0.018, and reported being less likely to use SM for friend and family connection, r (130) = −0.207, *p* = 0.018, access news, r (130) = −0.209, *p* = 0.017, follow pop culture, r (130) = −0.196, *p* = 0.026 and entertainment, r (130) = −0.214, *p* = 0.014. They were also less likely to follow a specific health figure or entity: health influencers, r (130) = −0.196, *p* = 0.026, health organizations, r (130) = −0.187, *p* = 0.033, health communities, r (130) = −0.186, *p* = 0.035, some other health account, r (130) = −0.198, *p* = 0.024. Those with a higher education level were more likely to use “X”, r (130) = 0.19, *p* = 0.031 while parents/caregivers were less likely to use “X”, r (130) = −0.034, *p* < 0.001. Furthermore, there was a very strong correlation between the use of Instagram and the likelihood of using social media to connect with family and friends, pop culture, news and entertainment, as well as following a health-related person/entity (influencer, organization, community other account) on social media. Lastly, those who used WhatsApp were more likely to use Snapchat and Facebook, r (130) = 0.218, *p* = 0.013 and r (130) = 0.287, *p* = 0.001, respectively.

The next set of correlation analyses (see Table 3) explored associations between healthcare and health information seeking patterns within certain demographics. Spanish speakers were less likely to consider a treatment or medicine, r (130) = −0.192, *p* = 0.028. No correlation was found between language or being a parent/caretaker and any social media platform or account. Older individuals were less likely to understand and relate with health information on social media, r (130) = −0.210, *p* = 0.016 and r (130) = −0.211, *p* = 0.016, respectively. They were also less likely to report that health information from SM helped them to make health-related decisions, r (130) = −0.221, *p* = 0.016. Females were more likely to have a primary care physician, r (130) = 0.281, *p* = 0.001, have annual healthcare appointments, r (130) = 0.249, *p* = 0.004, talk to the doctor about what they saw on social media, r (130) = 0.175, *p* = 0.046 and look up information after the doctor’s visit, r (130) = 0.285, *p* = 0.001. They were also more likely to use SM to gain health information, r (130) = 0.207, *p* = 0.018, finding it relatable, r (130) = 0.203, *p* = 0.021, and finding it helpful to make a health decision, r (210) = 0.222, *p* = 0.011.

Those with higher education were more likely to have a primary care physician, r (130) = 0.176, *p* = 0.045 but also reported having to wait beyond 2 weeks for an appointment, r (130) = 0.274, *p* = 0.002They were also more likely to attribute the delay in making an appointment to financial reasons, r (130) = 0.224, *p* = 0.011. Employed participants were more likely to use social media for health information to make a healthcare decision, r (130) = 0.188, *p* = 032, to delay an appointment due to finances, r (130) = 0.310, *p* < 0.001 and to have a chronic disease, r (130) = 0.187, *p* = 0.033. Having healthcare insurance was associated with being more likely to attend annual medical visits, r (130) = 0.237, *p* = 0.007, having a PCP, r (130) = 0.340, *p* < 0.001 and waiting 2 weeks or more to receive an appointment, r (130) = 0.350, *p* < 0.001.

When exploring associations between healthcare variables, social media use for health information and demographic variables, we found a positive correlation between having a PCP and having seen a doctor in the past year, r (130) = 0.563, *p* < 0.001. In addition, those who had a PCP and had received care in the past year were more likely to use social media to seek health information, r (130) = 0.263, *p* = 0.003 and r (130) = 0.297, *p* = 0.001, respectively. Both groups were also more likely to find the social media health information content relatable, r (130) = 0.275, *p* = 0.002 and r (130) = 0.287, *p* = 0.001, respectively, to talk to their doctors during the visit about their social media search, r (130) = 0.345, *p* < 0.001 and r (130) = 0.267, *p* = 0.002, respectively, and to seek information after the visit with a healthcare provider, r (130) = 0.325, *p*< 0.001 and r (130) = 0.289, *p* = 0.001, respectively. They were also more likely to decide on treatment, r (130) = 0.206, *p* = 0.018 and r (130) = 0.173, *p* = 0.049, and to feel that social media information influenced their decision-making, r (130) = 0.251, *p* = 0.004 and r (130) = 0.271, *p* = 0.002, respectively.

Furthermore, those who had a PCP and accessed care in the past year were more likely to make healthcare decisions based on the health information they saw on social media, r (130) = 0.217, *p* = 0.013 and r (130) = 0.180, *p* = 0.040, respectively. They were also more likely to have to wait at least 2 weeks before receiving an appointment, r (130) = 0.379, *p* < 0.001 and r (130) = 0.352, *p* < 0.001, respectively. Participants with a PCP were more likely to understand health information found on social media r (130) = 0.218, *p* = 0.013 and to have a chronic disease, r (130) = 0.209, *p* = 0.017. Those who had to wait at least two weeks for an appointment or delayed their appointment for financial reasons were more likely to also look up health information they might have heard about from their provider after their medical visit, r (130) = 0.243, *p* = 0.005 and r (130) = 0.186, *p* = 0.034, respectively.

In the final correlation analyses (Table 4) we explored associations between social media platforms, types of accounts followed and health information seeking behaviors. The only social media platform for which *several* statistically significant positive correlations with health information behavior was found is “Snapchat.” Its users were more likely to look up information on social media after a doctor’s visit, r (130) = 0.189, *p* = 0.031, to make health-related decisions based on the content they saw, r (130) = 0.188, *p* = 0.032, and to be influenced to consider a medication or treatment, r (130) = 0.199, *p* = 0.023. TikTok users were more likely to use health information from social media to decide on a specific treatment or medication, r (130) = 0.272, *p* = 0.002. Being an Instagram user was inversely correlated with easily finding health information on social media, r (130) = −0.174, *p* = 0.048, finding it easy to understand, r (130) = −0.255, *p* = 0.003, to relate to r (130) = −0.214, *p* = 0.015 and thinking that what was seen influenced their health decisions, r (130) = −0.221, *p* = 0.012.

Lastly, those who “followed” any of the 4 types of health accounts (health influencers, health organizations, health community or other health account) were less likely to understand social media health information -r (130) = −0.25, *p =* 0.004; r (130) = −0.249, *p* = 0.004; r (130) = −0.248, *p* = 0.004; r (130) = −0.256, *p* = 0.003, respectively, to find it relatable r (130) = −0.209, *p* = 0.017; r (130) = −0.193, *p* = 0.027; r (130) = −0.184, *p* = 0.036; r (130) = −0.217, *p* = 0.013, and to feel that it influenced their health decisions −r (130) = −0.219, *p* = 0.012; r (130) = −0.205, *p* = 0.020; r (130) = 0.019; r (130) = −0.205, r (130) = −0.196, *p* = 0.026.

### 3.4. Qualitative Results

All participants (N = 7) in the qualitative discussions were female and the average age was 31.14 (ranging from 30 to 35 years old). Most identified themselves as Hispanic/Latino. All participants had at least a high school diploma or GED. Participants discussed their use of social media and what they experienced when looking for health information on social media. More specifically, they were asked if the information they found was easy to understand and relate to and what type of information they were more likely to implement or consider using. Themes (shown on Table 5) describe patterns of social media use (including choices of platforms and types of accounts), participants’ digital and health literacy—the ability to find and understand health information—and the use of social media for health decision-making.

### 3.5. Social Media Use

All participants used some type of SM account, with the most used platforms being Instagram, TikTok, WhatsApp and YouTube. Most agreed to using SM on a regular basis several hours a day but doing so in intervals spread across the day. Everyone initially said they used social media to follow friends and family but then also admitted to using it to access the news and other information. In regard to the format of the platform, there was a preference for TikTok because of the accessibility of the content. Participants also reported sharing the health information they found on social media with family members. The type of information they shared consisted of details that they found helpful in their own decision-making leading them to want to pass it along. In the context of sharing, the use of the WhatsApp platform as a means of sharing health information was referred to in a derisive manner.

### 3.6. Finding Health Information

Several participants said that they prefer searching for information on social media more than on Google. This was especially the case for TikTok users. They attributed their preference to ease of scrolling through the content of the messages. They also felt it was easier to ask questions on TikTok than on Google. However, a few still preferred using Google instead of social media platforms when searching for health information. In the quantitative data, TikTok was significantly correlated with making health decisions about medication, but not necessarily health information.

Participants also shared what types of accounts they preferred to follow and why. Everyone followed at least one health-related influencer, and some also followed health organization. Others said they did not even realize health organizations had accounts, and one participant stated that she used TikTok the most. She also added that most organizations had an Instagram account but no TikTok accounts. Considering the political context of TikTok and its affiliation with China (and the recent narrative around this), understanding how active health organizations are on the platform would provide interesting insight. On the flip side organizations should recognize that individuals are using the platform for health information. In addition, almost every participant sought information for themselves and for family members. They were looking for more explanations about certain concepts and wanted to be able to make their own informed choice instead of being told what they should do.

### 3.7. Understanding Health Information

In understanding health information on social media, the problem wasn’t necessarily the content but rather the amount of information on a topic and then having to determine whether it is reliable and accurate information. Participants found the process overwhelming.

### 3.8. Using Health Information

Participants noted that they were more likely to use the information if there was a clear call to action. Another factor influencing the use of information gained from SM seemed to be relatability. If the content they saw was relatable and they could see themselves engaging in the proposed behaviors, they were more likely to use the information. There was also an element of trust. If they trusted the source of information, they were more likely to use it. Trust and response to a call to action was also associated with relatability. The frequency with which participants heard a consistent message also increased their trust in the accuracy of the message. The effect of algorithms was evident, especially on TikTok’s “for you page” (FYP), as confirmed by one of the participants who accessed more baby health content after looking up remedies for colic.

## 4. Discussion

As society has become increasingly digitally dependent, the use of social media has reached such popularity that it is now an integral part of everyday life. The COVID-19 pandemic and the influx of information and misinformation online seemed to have been the catalysts that encouraged many organizations and institutions to establish a social media presence. The purpose of this study was to explore the use of social media by an under-resourced population and the nature of their engagement with social media as a source of health information.

If there was ever a doubt that social media is widely used for health information in this population, participants (while overall on the younger side) in this study proved just that. Over 60% had accounts on three or more social media platforms and most of them used these daily. This was not limited to any particular type of account, as we found a strong, positive association of SM use for health information across all the categories of accounts (influencers, organizations and community). Although the use of the internet as a source of health information has been previously reported our findings support this to be true specifically in the context of social media [22,32].

Both the quantitative and qualitative results showed that users preferred content from influencers and most did not seek or follow health organizations or what would be considered a typical reputable, trusted source, such as the Centers for Disease Control and Prevention (CDC) or a local county health department. Most often content from these organizations tends to be ‘professional’ and ‘over-produced’ with graphics that are often hard to comprehend without studying them and thus can seem out of touch (too “perfect”, formal and not applicable to the audience’s reality) limiting broader engagement. We found, from our participants, that relatability is more valued and is what fosters trust by our participants and is more valued. FG participants said they liked hearing from people that they could relate to and that they could see themselves in. It is therefore important to understand that the appeal of the message may have as much to do with the way content is delivered as with the importance and relevance of the information. Therefore, the influencer becomes central to the message. When looking at the descriptive information on overall social media use, more than half of participants followed influencers and only to a limited degree “trusted” organizations that shared health information. In our qualitative discussions however, everyone looked to influencers for health information, whether or not they followed a “trusted” health organization. Again, further exploration could provide valuable insight in understanding if this is due to the format of messaging (less formal style of post, maybe a video which is not “overproduced”), since the only difference found between account types was in reach (number of individuals who saw the content).

Findings from this study confirm the widespread use of health information shared and consumed across all social media platforms, even though format, type of content and frequency may vary by audience. Based on the feedback from the discussions about social media posts, it seems that users seek information that is easily “digestible”, clear, using an “open” informative style rather than a “directive” style of communication (one that tells them what to do with no explanation of the reason behind the request). This points to the importance of social media as a tool to empower individuals to make informed decisions that are best for themselves and their families but also calls on the professional community to learn to more effectively execute this engagement.

Participants clearly used SM for health guidance, by seeking information that they considered to be factual and unbiased. Social media allowed them the distance to seek questions without judgment, thus preserving their dignity and self-respect. Of note, few participants mentioned the use of SM from the usual trusted sources, a finding supported by Kwame and Petrucka [33]. This prompts the question of whether messages from trusted sources are truly created to engage this population and points to an urgent need to explore how to best address this shortcoming.

The strong dependence on social media as a source of health information was seemingly of equal importance as the information provided to them by their healthcare providers. The fact that individuals are communicating with their providers about content from social media and are following up on provider guidance on social media makes the case for social media being a factor in health literacy and influencing health behaviors.

Of note, after the peak of the COVID-19 pandemic crisis, the usual wait for visits had increased to 38 days [34,35]. Those who had to wait at least two weeks for an appointment or experienced a delay due to financial reasons were more likely to go to social media for health information about something that was discussed with their health provider. The significant positive association with appointment delays and delays for financial reasons with talking to a doctor about HI on SM suggests that, even after waiting to see their healthcare providers, participants use social media to corroborate what they have been told during the visit. They may do so to ensure their wait and effort was worthwhile, which is in keeping with the increasing trends to self- and co-diagnosing [36,37,38]. This may also be an indication of lack of trust among those with lower incomes as there is a history of hesitancy among those most disenfranchised by society [39].

Considering that the topic of interest is social media, it is not surprising that different generations and groups of people use SM in different ways. The younger the individual, the better their understanding of what was viewed on social media and the more relatable and useful they found it in making health-related decisions. The average age of those agreeing to participate in the qualitative data collection (interviews) was slightly younger than that of those who participated in the quantitative data collection (31 years vs. 34 years of age) and only females participated in further discussions after completing the survey. This is not surprising as the demographic variable that had the most significant associations across social media variables was gender. Women were more likely to have a PCP and to attend their yearly visits. They were also more likely to share the information from social media and to search for health information after their medical visits. In other words, they combined social media findings with information garnered during the medical appointments to arrive at a decision. This also is not surprising considering women tend to be more invested in their health and are often the ones making the health-related decisions for their families [28,40].

The inverse correlation between Instagram use and the ability to find, relate with and understand information (health literacy) is somewhat unexpected and concerning, especially since a high percentage of our study participants use Instagram. Interestingly, there was not necessarily a preference for any particular category of account (health organization or influencer). So, these results are not due to the type of account used to provide information. A possible reason may be due to the characteristics of individuals using Instagram such as low literacy.

Or this could be the result of participants reaching a point of information overload, a phenomenon that was mentioned in our qualitative discussions and was a key component of the theme that relates to understanding of health information. Participants stated that they saw so much from so many sources about the same topic that they were overwhelmed. Since many have different types of accounts it could be that there is a point where access to information becomes too much and results in information overload [19,41]. The lack of understanding might not necessarily refer to individual pieces of content, but rather the overall intake of information from social media about a particular topic. Further exploration of these correlations is necessary to draw any meaningful conclusions.

Most participants who used TikTok admitted to it being the first stop when searching for information. This is supported by data speaking to the increasing popularity of Tik Tok [42]. TikTok’s unique algorithm and platform design seems to have been effective in not only attracting users but creating a dependency, of it competing with Google in information searches. Some preferred it over other platforms because of the anonymity it provided. In recent versions of the app, users must create an account to view content, but they can connect with other users and can remain relatively anonymous, even if they want to engage with content through comments.

Another stated reason for preferring TikTok was the format of the content. The platform has undergone several changes from its initial rule that content could only be viewed in 15 s and supported only “short form” content (short messages). It has since been adapted for “long form” content which lasts up to 10 min. In addition, it has recently also become an “influencer” platform.

Since participants in our qualitative discussions were either a parent or caretaker, they appreciated being able to scroll for a few minutes and go through several pieces of content in short periods of time. When searching for specific information, they liked being able to scroll through videos from several accounts instead of having to click through websites and pause to read content, something that would be necessary on Google. Interestingly, those seeking health information on TikTok did so seeking explanations to questions they had to help them make informed decisions.

The ability to multitask and still gain knowledge during short periods of time is another perceived advantage of TikTok. Its change in algorithm and video experience resulted in its increasing popularity and, as a result, several other platforms made similar changes: Meta (another name for Facebook apps) overhauled its platform and algorithm to account for reels, YouTube introduced Shorts and even LinkedIn developed its own version of short interactions.

The most unexpected result was the positive association between Snapchat and various health information-seeking behaviors, especially for decision-making. Snapchat was the only platform that was directly associated with decision-making related to health. The platform is not typically considered a resource for information but has evolved to incorporate that element with many organizations, news sites and podcasts using an informational voice. There are also several health influencers that have a considerable number of following on the platform and even the CDC regularly shares content there. Researchers are also considering the use of this platform as a health education tool especially for younger users [43]. As such it should be further explored.

On social media, health information can be accessed passively or actively. Active forms imply engagement from recipients and can include looking up a specific topic in a search on a platform or looking up a “creator” that shares information within a specific genre. Passive content is limited to posts that show up on a “feed” from accounts one already follows. Our paper did not distinguish between the two forms. This is important to understand this limitation as SM is driven by one’s own pattern of use: as one uses SM, more information of a similar type appears. In addition, a factor that was not explicitly shown in the data but is a clear mediating factor is the use of algorithms that SM platforms use to engage users. Depending on one’s searches and engagement with content, the algorithm usually offers similar types of health contents which is especially true on the TikTok “for you page” (FYP). Thus, it takes more than just creating a well-produced message by trusted sources. The complexity of the algorithm, which is partially influenced by the likelihood of viewer engagements, must be taken into account if the creator of the message is to reach its targeted audience.

One would have expected that having a consistent primary care provider would have provided enough trust that patients would be less inclined to rely on social media to make decisions regarding their health, but this was not the case. Interestingly, patients who had a PCP and kept their annual medical appointments were more likely to use social media. Not only does this indicate a strong intersection between healthcare access and digital literacy in general (including navigation), but it also confirms the important place that social media information has in health decision-making of those who regularly visit their healthcare providers [36].

**Figure 4:**
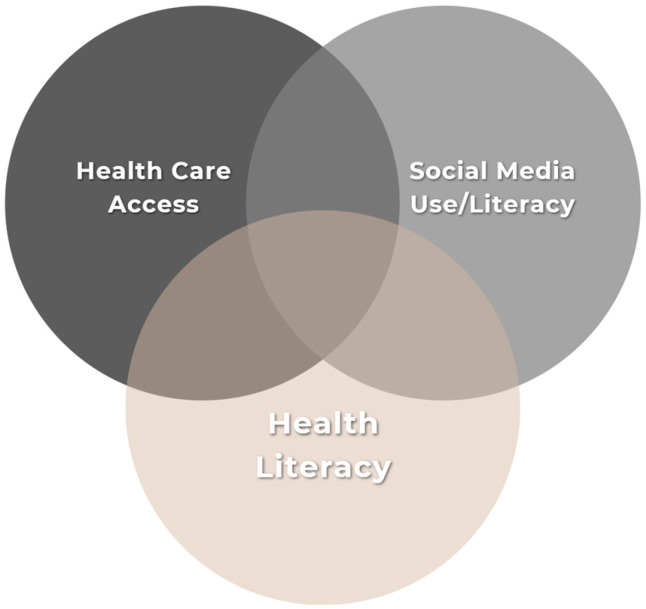
Graph depicting intersectionality between health literacy, access to healthcare and social media use.

The correlation between healthcare access, understanding and using health information and using social media found in the quantitative results, suggests an interrelatedness of these concepts, as shown in Figure 4, that influences how health information on social media is received by audiences. Those who had a PCP and attended their annual visits also reported digital health literacy indicating that for them healthcare information on social media was easy to find, understand and relate to. They also reported that they used what they learned on SM to inform their health decisions and to make treatment and medication-related decisions.

While our data did not allow us to make assumptions about the veracity about the information respondents find, it suggests that having access to social media may improve overall health literacy as individuals use social media to make their health decision and follow up on provider appointments. Not only are social media users finding health information on social media, they are being influenced by that information and even turning to social media to further expand on what they hear from their providers. It is imperative for health professionals to recognize how social media use influences health conversations and behaviors. For those who worry about misinformation on social media, the fact that our respondents mainly used it to follow up on provider recommendations is encouraging. Future research should explore the interrelatedness of access to healthcare, social media use and health literacy as well as how this is reflected in specific platforms among specific populations, especially those most under-resourced. In addition, there should be a better understanding of why patients feel the need to turn to social media after seeing their provider and how providers can supplement their interactions which are most often time limited, with more intentional referrals to social media that ideally would combine the relatability users seek with content quality control.

All of the findings from this study are important for health organizations to consider when creating content especially since these can influence the algorithm and thus determine the reach and the effectiveness of their messages. Engaging individual creators who understand what it takes to reach a specific audience when sharing social media content may be a valuable investment for health organizations that are serious about effectively communicating accurate health information to under-resourced populations

## 5. Strengths and Limitations

It is important to note the study’s limited qualitative sample size, the fact that it used a regional convenience sample to recruit lower-income participants, its ratio of males to females and the relatively young age of our participants as a limitation. A larger, more balanced gender distribution may have lent itself to further insights, especially for males and our results cannot be generalized to other populations. The timing of the study may have also influenced recruitment as it took place during election season and during the change in the US government, a time with much uncertainty and a tense social and political climate. This may have been the reason why Spanish participants requested individual interviews rather than participation in a focus group. However, given the lack of available published studies about SM use and health information, we felt that our small mixed methods study was an acceptable way to begin to further explore this new area of research to better understand health information seeking patterns on social media in a region with under-resourced populations. As stated previously, this study was intended to explore the topic and be a pilot for future studies which should also evaluate the accessibility and organizational health literacy of health information on social media in the context of current algorithms and then delve into the impact on behavior change, perhaps in a longitudinal fashion. More information may have also helped determine which variables should be controlled for. However, social media platforms change so quickly it is difficult to apply the same methodological model over an extended period of data collection, which is why a mixed-methods cross-sectional design was determined for this study. Although we had enough power for this study, participants’ assessments occurred at two different times, and this may have affected the results.

## 6. Conclusions

It is evident that social media is widely used across many populations, including those living in an under-resourced region and that it has become an important source of health information for many users. However, there seems to be a “disconnect” between social media created by established credible sources of health information and the patterns of social media use among under-resourced populations, a group mainly exposed to non-organizational “influencers.” The results of this study could inform health organizations on the best ways to select platforms based on demographic characteristics, and more effectively connect with their audiences for improved health messaging to increase patient access to accurate health information. Future research built on this study should explore specific messaging on specific platforms among under-resourced communities. This can not only inform best practices, but also policies around health information and mitigating misinformation in the era of AI and “Do your own research.”

Given that our respondents reported using SM after healthcare visits to deepen the information they received for health decision-making, it is critically important for health organizations to provide engaging and accurate content to empower the consumers to help them make informed decisions and communicate more effectively with their healthcare providers. While our findings could provide critical information to healthcare entities to guide their social media reach, future research should extend this avenue of inquiry, especially considering the increasing burden on an understaffed healthcare system to provide quality care. This in turn could lead to more health literacy, a needed step towards implementing favorable health behaviors and ultimately result in better community health outcomes.

## Figures and Tables

**Table 1:** Demographic and healthcare descriptives.

Demographic Charaeristic/Variable	Description	*n*	%
Age (Mean) = 34 (SD = 20.97)			
Preferred Language	English	206	80.15
	Spanish	50	19.46
Gender	Male	32	12.45
	Female	222	86.38
	Non-Binary	2	0.78
Parent/Caretaker	No	97	38.13
	Yes	158	61.09
Education	Less than High School	27	10.51
	High School or GED	75	29.18
	Certificate or Trade School	24	9.34
	Bachelor’s Degree	23	8.95
	Graduate Degree	16	6.23
Employment	Unemployed	82	31.91
	Part Time	33	12.84
	Full Time	128	49.8
	2 or more jobs	11	4.28
Health Insurance	No Insurance	31	12.01
	Private	128	49.81
	Public/Government	97	37.74
Saw a doctor in the past year	No	33	12.8
	Maybe	9	3.5
	Yes	211	82.1
Had a primary care physician	No	50	19.4
	Maybe	28	10.9
	Yes	177	68.9
Had a diagnosed chronic condition	No	162	63
	Maybe	20	7.8
	Yes	72	28
Waited two or more weeks for an appointment	No	67	26
	Maybe	23	8.9
	Yes	165	64.2
Delayed appointment for financial reasons	No	112	43.6
	Maybe	31	12.1
	Yes	111	43.2

**Table 2:** Bivariate correlation analyses between social media platforms, accounts, uses and demographic variables.

	Language	Age	Gender	Parent/ Caretaker	Education	X	Snapchat	WhatsApp	TikTok	Facebook	Instagram
X	−0.202 *	−0.025	−0.119	−0.34 ***	0.190 *	1	0.367 ***	0.118	0.270 **	0.157	−0.028
Snapchat	−0.166	−0.065	0.037	−0.154	0.002	0.367 ***	1	0.218 *	0.286 ***	0.104	−0.046
WhatsApp	0.287 ***	−0.098	0.080	−0.041	0.077	0.118	0.218 *	1	0.075	0.287 ***	−0.074
TikTok	−0.157	0.072	0.040	0.060	−0.052	0.270 **	0.286 ***	0.075	1	0.228 **	−0.115
Facebook	0.149	−0.032	−0.070	0.040	−0.101	0.157	0.104	0.287 ***	0.228 **	1	−0.125
Instagram	−0.065	−0.114	−0.207 *	−0.132	−0.098	−0.028	−0.046	−0.074	−0.115	−0.125	1
Friends/ Family	−0.053	−0.113	−0.207 *	−0.123	−0.103	−0.038	−0.054	−0.078	−0.121	−0.130	0.999 ***
Pop Culture	−0.100	−0.117	−0.196 *	−0.146	−0.070	0.008	−0.012	−0.069	−0.108	−0.153	0.988 ***
News	−0.063	−0.112	−0.209 *	−0.138	−0.088	−0.024	−0.048	−0.067	−0.115	−0.124	0.997 ***
Entertainment	−0.085	−0.111	−0.214 *	−0.133	−0.088	−0.008	−0.036	−0.083	−0.117	−0.143	0.996 ***
Health Orgs	−0.056	−0.112	−0.187 *	−0.128	−0.090	−0.038	−0.064	−0.062	−0.121	−0.136	0.994 ***
Health Influencers	−0.052	−0.119	−0.196 *	−0.129	−0.099	−0.024	−0.043	−0.068	−0.111	−0.125	0.996 ***
Health Comms	−0.069	−0.113	−0.186 *	−0.118	−0.099	−0.028	−0.040	−0.087	−0.112	−0.127	0.990 ***
Other Health Accounts	−0.043	−0.111	−0.198 *	−0.114	−0.103	−0.047	−0.044	−0.069	−0.132	−0.132	0.989 ***

All values in bold were statistically significant. Significance indicated as follows: * Correlation is statistically significant at *p* < 0.05. ** Correlation is statistically significant at *p* < 0.01. *** Correlation is statistically significant at *p* < 0.001

**Table 3:** Bivariate correlations between healthcare access and health information on social media with demographic variables.

	Language	Age	Gender	Parent/ Caretaker	Education	Employment	Insurance	Healthcare	PCP	Chronic Disease	Appt Wait	$ Delay
Looked up HI on SM	0.118	−0.062	0.207 *	−0.033	0.129	0.081	0.078	0.297 ***	0.263 **	0.095	0.060	0.075
Made a health decision after seeing HI on SM	−0.099	−0.103	0.090	−0.034	0.160	0.188 *	0.056	0.180 *	0.217 *	0.083	0.096	0.139
HI easy to find on SM	−0.074	−0.105	0.070	−0.082	0.044	0.098	0.030	0.108	0.161	0.056	−0.145	0.006
Understood HI on SM	0.016	−0.210 *	0.054	−0.081	0.007	0.055	−0.083	0.112	0.218 *	−0.011	−0.057	0.054
Found HI on SM relatable	−0.093	−0.211 *	0.203 *	0.039	0.082	0.044	0.047	0.287 ***	0.275 **	−0.054	0.159	0.161
HI on SM influenced health decision	−0.018	−0.221 *	0.222 *	0.117	0.001	−0.074	0.023	0.271 **	0.251 **	−0.009	−0.034	−0.001
Talked to doctor about HI on SM	−0.078	−0.082	0.175 *	0.109	−0.124	−0.009	0.079	0.267 **	0.345 ***	0.063	0.081	−0.031
Looked up HI on SM after talking to doctor	−0.042	−0.127	0.285 **	−0.049	0.014	0.153	0.063	0.289 ***	0.325 ***	0.073	0.243 **	0.186 *
Considered treatment or medicine after seeing HI on SM	−0.192 *	−0.114	0.127	0.041	0.041	0.162	0.077	0.173 *	0.206 *	−0.093	0.098	0.111
Healthcare: Saw a doctor in the last year	−0.022	−0.111	0.249 **	0.105	0.098	−0.065	0.237 **	1	0.563 ***	0.095	0.352 ***	−0.093
Has a primary care physician (PCP)	0.004	−0.095	0.281 **	0.013]	0.176 *	0.013	0.340 ***	0.563 ***	1	0.209 *	0.379 ***	−0.150
Has a chronic disease	0.082	0.012	0.076	−0.072	0.094	0.187 *	0.140	0.095	0.209 *	1	0.204 *	0.086
Appt wait: Waited >2 weeks for appointment	0.011	−0.093	0.138	0.004	0.274 **	0.072	0.350 ***	0.352 ***	0.379 ***	0.204 *	1	0.163
$ Delay: Delayed an appointment for financial reasons	−0.013	−0.060	0.032	−0.130	0.224 *	0.310 ***	0.032	−0.093	−0.150	0.086	0.163	1

All values in bold were statistically significant. Significance indicated as follows: * Correlation is statistically significant at *p* < 0.05. ** Correlation is statistically significant at *p* < 0.01. *** Correlation is statistically significant at *p* < 0.001.

**Table 4:** Bivariate correlation analyses between health information and social media variables.

	Looked Up HI on SM	Made a Health Decision After Seeing HI on SM	Felt It Was Easy to Find HI on SM	Understood the HI Seen on SM	Found HI on SM Relatable	HI on SM Influenced Health Decisions	Talked to Doctor About HI on SM	After Talking to Doctor Looked Up HI on SM	Considered Treatment/Med After Seeing on SM
X	0.039	0.142	−0.063	0.049	0.053	−0.039	−0.006	0.112	0.137
Snapchat	0.124	0.188 *	−0.011	−0.055	0.100	0.105	0.068	0.189 *	0.199 *
WhatsApp	0.048	0.088	−0.039	−0.026	−0.004	0.020	−0.008	0.110	0.155
TikTok	0.092	0.084	0.017	0.094	0.083	0.126	0.046	0.117	0.272 **
Facebook	0.100	0.017	−0.085	0.053	0.092	0.064	0.117	0.163	0.160
Instagram	−0.164	−0.108	−0.174 *	−0.255 **	−0.214 *	−0.221 *	−0.136	−0.140	−0.111
Health Organizations	−0.141	−0.101	−0.154	−0.25 **	−0.209 *	−0.219 *	−0.118	−0.141	−0.105
Health Influencers	−0.138	−0.096	−0.164	−0.249 **	−0.193 *	−0.205 *	−0.113	−0.132	−0.094
Health Communities	−0.135	−0.088	−0.16	−0.248 **	−0.184 *	−0.205 *	−0.091	−0.124	−0.081
Other Health Accounts	−0.135	−0.098	−0.156	−0.256 **	−0.217 *	−0.196 *	−0.133	−0.124	−0.105

All values in bold were statistically significant. Significance indicated as follows: * Correlation is statistically significant at *p* < 0.05. ** Correlation is statistically significant at *p* < 0.01.

**Table 5:** **Key informant interviews** (KII) and focus group (FG) quotes.

Theme	Quote
**Social Media Use**	*“So he just does like the really short, like reels that I feel like are easy to so like. Oh, I can take that and do that, and that’s something easy for me.”*—FG Participant *“I think for me, social media is where I see most of it. ‘cause I I don’t ever really go on TV to click on the news or anything like that, but like when there’s like a new outbreak or like a recall on something I feel like it’s mostly on social media where I’m finding out about these things.”* —FG Participant *“I just you know, I just started like sharing and reposting things of help, you know, being able to help other people when I would see like help, because I feel like, maybe that’s something that I was looking for, … So I do, you know now, occasionally like to post like we post stuff like that? Yeah.”* —KII Participant *“Oh yeah, my mom, she always sends me stuff on WhatsApp”*—KII Participant
**Finding Health** **Information**	*“Actually, you know, it’s it’s it’s weird. Because if you would ask me like, maybe a year ago, I would have say, Google, I just Google it. I just go. And now, because of the I don’t know, I thought it was because of the time. But I found that like, there’s a lot of people that do it. I’m like, Okay, I’m not alone. I just put it like on TikTok, like how to make even on YouTube I used to. That’s why I say right now, occasionally, … And then it’ll give me like an hour long, other than like, you know, comparing to TikTok that we have, or like the shorts on YouTube shorts that it’s very quick by like a minute. You know the whole information, the whole recipe. So now I look it up more on social media. Yeah.”*—FG Participant *“Yes, I will look at TikTok information from them, and then I will Google it. And then sometimes I would find, like the same exact thing that TikTok is talking about. But if it wasn’t for TikTok I wouldn’t be able to be able to Google this stuff.”*—KII Participant *“I feel like with TikTok. Not a lot of those big organizations like have their own profile, because TikTok is kind of more of a different type of platform.”*—FG Participant
**Understanding Health Information**	*“Yeah, you know, it’s actually not something, not that helpful. Because last time I was reading all like, Oh, it’s really good to eat before you know your 1st meal. And then I came across another Tik Tok. It was actually a Tik Tok, and it was another mom. and it was like the opposite, like you do not want to do this. And I was like, Oh, man!”*—FG Participant *“I think, depending on what it is like. Sometimes it is helpful, and then sometimes I think I think it is specific to what, to what you’re looking at, because I feel like some topics. There’s a lot of different opinions and perspectives on it. So I feel like it takes more research like to find out like which, what?”*—FG Participant *“You know how many people are saying this, and then the different types of people that are saying it and who it’s coming from. And for me, I think it’s a mix of like people who have some kind of like professional background in it. And then also, people who have some kind of personal experience in it.”*—FG Participant
**Using Health Information**	*“… but I have like an example would be when she was a newborn should always have. I believe you pronounce it colic. And so I would always search up like massages I could do on her, or how other parents would relieve the pain for them. So I’ve done it as well for her.”*—FG Participant *“… like I like seeing like real people going through that. So I think if it’s something relatable or I don’t know, like I think, when the COVID vaccine was going around, and people were like hesitant on getting or not. I I like seeing like. Oh, well, I got the COVID vaccine and yes, I did get COVID, but you know I didn’t get as sick because I had the vaccine.”*—FG Participant *“I think that when I do like, if I if I intentionally search something like on my symptoms or something I won’t ever take the 1st video, like I take it, with a grain of salt. And then, as as like, they said, right now, I think the more that you start seeing like the same type, the more you start kind of believing it. So I I think it depends to who who’s giving you this information.”*—FG Participant

## Data Availability

The data presented in this study are available on request from the corresponding author due to privacy.
